# Genetic Diversity and Population Genetic Structure of a Guzerá (*Bos indicus*) Meta-Population

**DOI:** 10.3390/ani11041125

**Published:** 2021-04-14

**Authors:** Maria Gabriela C. D. Peixoto, Maria Raquel S. Carvalho, Andrea A. Egito, Raphael S. Steinberg, Frank Ângelo T. Bruneli, Marco Antônio Machado, Fernanda C. Santos, Izinara C. Rosse, Pablo Augusto S. Fonseca

**Affiliations:** 1 Embrapa Gado de Leite, Juiz de Fora 36038-330, Brazil; frank.bruneli@embrapa.br (F.Â.T.B.); marco.machado@embrapa.br (M.A.M.); 2 Departamento de Biologia Geral, Instituto de Ciências Biológicas, Universidade Federal de Minas Gerais, Belo Horizonte 31270-901, Brazil; mraquel@icb.ufmg.br (M.R.S.C.); raphael.steinberg.ds@gmail.com (R.S.S.); fernandacsantos.bio@gmail.com (F.C.S.); izinara.rosse@gmail.com (I.C.R.); pfonseca@uoguelph.ca (P.A.S.F.); 3 Embrapa Gado de Corte, Campo Grande 79106-550, Brazil; andrea.egito@embrapa.br; 4 Centre for Addiction and Mental Health (CAMH), Campbell Family Mental Health Research Institute, Toronto, ON M5T 1R8, Canada; 5 Departamento de Farmácia, Escola de Farmácia, Universidade Federal de Ouro Preto, Ouro Preto 35400-000, Brazil; 6 Centre for Genetic Improvement of Livestock, Department of Animal Biosciences, University of Guelph, Guelph, ON N1G 2W1, Canada

**Keywords:** Zebu cattle, genetic variability, artificial selection, sustainable management, livestock conservation

## Abstract

**Simple Summary:**

The Guzerá breed is one of the most relevant animal genetic resources for tropical and extreme environments, that is, low and high temperatures, and dry or humid environments. These animals were selected for beef, milk, or dual-purpose (beef and milk), and were extensively used to produce crossbred animals. Consequently, subjecting the breed to intense genetic bottlenecks in Brazil. The local scientific community and breeders have pursued a sustainable management and conservation program over the last 50 years. To evaluate the efficacy of these efforts, we characterized genetic diversity and structure in a Guzerá meta-population. DNA samples of 744 animals from one dairy, nine dual-purpose, and five beef herds were genotyped for 21 microsatellite loci. The genetic diversity estimates suggested a low fixation index, revealing a large genetic diversity in Guzerá herds. The dual-purpose herds/selection lines are the most uniform subpopulation, while the beef one preserved larger amounts of genetic diversity, representing a genetic diversity reservoir for the breed. In addition, the dairy herd showed to be genetically distant from other herds. Taken together, these results suggest that this Guzerá meta-population has higher genetic diversity, a lower degree of population subdivision, and a lower inbreeding level.

**Abstract:**

The Brazilian Guzerá population originated from a few founders introduced from India. These animals adapted well to the harsh environments in Brazil, were selected for beef, milk, or dual-purpose (beef and milk), and were extensively used to produce crossbred animals. Here, the impact of these historical events with regard to the population structure and genetic diversity in a Guzerá meta-population was evaluated. DNA samples of 744 animals (one dairy, nine dual-purpose, and five beef herds) were genotyped for 21 microsatellite loci. Ho, He, PIC, F_is_, F_it_, and F_st_ estimates were obtained considering either farms or lineages as subpopulations. Mean Ho (0.73) and PIC (0.75) suggest that genetic diversity was efficiently conserved. F_it_, F_is_ and F_st_ values (95% CI) pointed to a low fixation index, and large genetic diversity: F_it_ (Farms = 0.021–0.100; lineages = 0.021–0.100), F_is_ (Farms = –0.007–0.076; lineages = −0.014–0.070), and F_st_ (Farms = 0.0237–0.032; lineages = 0.029–0.038). The dual-purpose herds/selection lines are the most uniform subpopulation, while the beef one preserved larger amounts of genetic diversity among herds. In addition, the dairy herd showed to be genetically distant from other herds. Taken together, these results suggest that this Guzerá meta-population has high genetic diversity, a low degree of population subdivision, and a low inbreeding level.

## 1. Introduction

Maximum conservation of genetic diversity is the best guarantee of long-time survival of a breed or a population under artificial selection. Significant loss of genetic variation due to Bulmer effect, inbreeding, and, especially in small populations, genetic drift in the populations under directional selection could imply in low response to genetic selection, decreasing genetic progress rate as well as adaptive performance [1,2,3]. On the other hand, the rescue of genetic variation by the strategy of introducing animals from other populations or other breeds is an attempt that could reduce the future genetic progress and disrupt the population phenotypic uniformity and adaptive performance. Many strategies were proposed to deal with this problem and the use of genomic tools has been presented as an opportunity to deal with sustainable genetic selection [4,5]. Therefore, monitoring genetic diversity is fundamental in any artificial selection process. The genetic conservation of local and adapted breeds is an important concern due to its contribution to current or future scenarios for genetic improvements and trait selection [5,6].

Guzerá (*Bos indicus*) cattle are originally from India, particularly from the dry lands of the Kankrej region. A relatively small population was brought from India at the end of the 19th century and expanded to Brazil, where it became adaptable to various, especially harsh, environmental conditions [7,8]. In Brazil, this breed has been selected for beef, milk, and, recently, dual-purpose (beef and milk) production. This breed remained prevalent in bovine livestock until the 1930s. By the middle of the last century, however, the Guzerá population decreased due to its extensive use in crossbreeding schemes [7,9]. The herdbook of the Guzerá breed was initiated in 1936 and closed in 1971. Subsequently, animals with an unknown pedigree but with the typical morphology of the Guzerá breed were registered in an “open herdbook”. A total of 211,107 animals were registered, with 84% in the closed herdbook. In 2013, 11% of males and 89% of females were registered, out of 17,295 animals born.

Analyzing genealogical data, Faria et al. (2009) [9] obtained an effective population size of 104 for all registered Guzerá individuals. A similar value (98) was calculated by Peixoto et al. (2010) [7] for Guzerá herds selected for milk purposes, suggesting genetic variability losses in this subpopulation.

In 1993, FAO included the Guzerá in the World List for Domestic Animals Diversity. This list includes breeds of economic, scientific, cultural, and agricultural interest to be conserved through management. Motivated by the importance of this breed as a genetic resource, two focused, interconnected breeding programs were initiated: one for milk traits (National Program for the Improvement of Guzerá for Milk Traits, PNMGuL) other for beef traits [10].

Nevertheless, because of the selection process based on genetic evaluation, further reduction in the number of sires used by breeders, due to the selection process based on genetic evaluation ranks, has become a concern. In the medium—and long-term, this could result in the reduced genetic variability of the population and increased levels of inbreeding [7,9]. From this perspective, better knowledge of the population structure and genetic variability within and among Guzerá subpopulations could be useful in future definitions of selection and mating strategies based on marker-assisted conservation [11,12,13,14].

Genetic diversity and population structure have been studied in bovine populations using microsatellite markers [15,16,17,18] or single nucleotide polymorphism (SNP) markers [19,20,21,22]. The genome of the Guzerá breed was published in 2016 and a large number of variants with putative functional impacts were described [23]. Moreover, the impact of the recent bottlenecks in the current genetic diversity estimates for the Guzerá were described using a genome-wide SNP genotyping approach [24]. However, the genetic composition within lineages, farms, and selection lines, as well as the genetic variability across the breed are still unclear, making the identification of genetic reservoirs and the implementation of genetic management harder. Microsatellite markers, specifically, have been largely used to estimate the magnitude of genetic variability in animal populations following FAO recommendations as well as population genetic structure [25].

The main objective of this study was to ascertain genetic diversity and population structure in a meta-population of the Guzerá breed. A meta-population was defined in the current study as the fraction of the population to which we had access, that is, the subpopulation made up of herds that take part in the breeding programs and among which some degree of gene flow occurs. From the results of this study, we expect to effectively contribute to the sustainability of this breed by minimizing the loss of diversity and allowing long-term genetic progress.

## 2. Materials and Methods

### 2.1. Sampling

This study used blood samples from 744 animals, representing around 10% of the animals in each of the 15 major Guzerá herds located in Minas Gerais state, in southeastern Brazil. The majority of herds are concentrated in the Minas Gerais State, which provides proven genetic material from Guzerá nationwide. Currently, some herds exploit Guzerá animals for dual-purpose (dairy and beef), using proven sires from both breeding programs. Other herds use Guzerá strictly for beef purposes (Supplementary Table S1). Currently, the breed is under a breeding scheme, and the use of AI, as well as the availability of sires with accurate genetic merit, has increased in the years since the release of the first sire summary for the breed [7,9]. 

A herd was defined as the group of animals present in a unique breeding location or farm. Many farms take part in the traditional breeding programs, owning herds composed of their own lineages or a mix composed of their own and external lineages from the whole Brazilian Guzerá livestock. Furthermore, the sampled herds have different selection objectives (selection lines), which were defined here as the group of animals selected for beef, dairy or dual-purpose. Two approaches were applied to understand the structure of this meta-population. In the first approach, each farm was considered a subpopulation; in the second approach, each lineage was considered a subpopulation. The comparison of genetic diversity among different lineages or farms was used to infer the population structure and gene flow in subpopulations.

### 2.2. DNA Extraction and Genotyping

Genomic DNA extraction, PCR amplification, and capillary electrophoresis were developed as described previously [26]. The microsatellites used in this study were chosen based on a set of loci, internationally recommended by both FAO and ISAG (International Society of Animal Genetics), to investigate the genetic diversity of animal species [27]. These microsatellites were identified from the bovine genetic linkage map available at MARC/USDA (Meat Animal Research Center/United States Department of Agriculture). A panel of 21 microsatellites (ILSTS093, MNB-208, BM1237, BMS2614, BM7169, RM150, BMS2252, JAB8, DIK5382, DIK4383, NRDIKM004, NLBCMK13, DIK4593, MNB-88, MNS-20, DIK5307, DIK5183, DIK4513, DIK2279, DIK5300, and DIK1143) was defined as the most informative, specifically for the Guzerá animals, based on their polymorphism information content (PIC).

### 2.3. Polymorphic Information Content and Average Exclusion Probabilities

Allele frequencies were extrapolated from direct counting. The number of alleles present in the population, numbers of individuals genotyped, expected (He) and observed heterozygosities (Ho), Polymorphism Information Content (PIC), and non-exclusion probabilities were calculated for each locus using the Cervus 3.0 [28] software and averaged for the 21 loci.

### 2.4. Global Hardy–Weinberg Tests

The Hardy–Weinberg equilibrium (HWE) was tested to support the assessment of the degree of allelic and genotypic diversity in the population. Departures from the HWE could indicate the impact of artificial selection (due to non-random mating) or reduced population size. Heterozygote deficiency was accessed using a multi-sample version of the Score test or U test with the GenePop 4.0 software [29]. The multi-sample score test of Rousset and Raymond (1995) is a global test across loci and/or samples performed using a Markov chain (MC) algorithm. The exact P-value of this test [30] was estimated through a 10,000 dememorization step, followed by 20 batches of 5000 iterations each. Two results were provided for each test by the MC algorithm: the estimated P-value associated with the null hypothesis of HWE, and the standard error (SE) of this estimate. The Hardy-Weinberg equilibrium was also tested using the Cervus 3.0 [28] software using a chi-square goodness-of-fit test.

### 2.5. F-Statistics and Genetic Distances

To estimate genetic differentiation and relationships among subpopulations, Wright (1978) [31] allele frequency-based correlations (individual fixation index (F_is_), fixation index (F_st_), overall fixation index (F_it_)) were calculated following the variance-based method of Weir and Cockerham (1984) [32], using the FSTAT (version 2.9.3.2) program [33]. Differentiation was estimated by averaging the 21 locus single estimates of those F statistics. Estimates were obtained using two approaches. In the first approach, the 21 loci were analyzed by grouping the subpopulations according to the sample collection farms, comprising 744 animals in 15 farms. This approach was designed to assess the influence of the geographical proximity of farms on the exchange of animals or the migration between them. In the second approach, the subpopulations were grouped according to the lineage or origin of animals. In this approach, lineages with a small number of individuals were removed from the analysis, and the final sample comprised 664 animals from 15 lineages. F_is_ and pairwise F_st_ values were calculated for each subpopulation using the Molkin v3.0 software [34]. The AMOVA algorithm, available in the ARLEQUIN computational package, was used to evaluate the partitioning of the genetic variance between different sources of variation and to test their significance using a non-parametric permutation approach [35].

Pair-wise standard genetic distances (D) [36] were calculated from allele frequencies and were used to build a dendrogram, summarizing genetic relationships using a neighbor-joining method [36] implemented in Poptree2 [2]. Bootstrap re-sampling (*n* = 1000) was performed to evaluate the robustness of the clusters. Additionally, a Neighbor-Net graph (Bryant and Moulton, 2004), based on the F_st_ estimated for lineages as subpopulations, was constructed using the SplitsTree4 [37] program.

The program structure was used to obtain the cluster assignment of lineages through a Bayesian approach, implementing the Markov Chain method with increasing numbers of inferred populations [38]. The tests were conducted assuming the admixture model based on the correlations between allelic frequencies and applying burn-in periods of 50,000 and 300,000 iterations for each dataset. Two to 17 inferred clusters were performed with three independent runs each. The determination of the real number of selection lines (K) was carried out by implementing the Evanno method [39] to assign lineages to each selection line, supposing some differentiation between them. The graphic display was made using the DISTRUCT software [40].

### 2.6. Quantification of the Contribution to Diversity

The contribution of each lineage to meta-population genetic diversity was assessed using the method proposed by Caballero and Toro (2002) [41] with the use of the Molkin v3.0 software [26]. With this method, the criterion was the maintenance of the maximum overall Nei (1987) [36] genetic diversity (GD) in the set of lineages.

## 3. Results and Discussion

### 3.1. Performance of the Microsatellite Panel

The microsatellite panel was evaluated in terms of the number of alleles, He, Ho, PIC, and non-exclusion probabilities, presented in Supplementary Table S2. About 74% (15) of microsatellite loci were highly polymorphic. The number of alleles present in each locus ranged from 8 to 27. Five loci had a high allelic richness (≥18) and the other 10 showed several alleles ranging between 11 and 15. This provides evidence for the existence of substantial genetic variability in the Guzerá population. Combined results, including the 21 loci, are presented in Table 1.

The mean observed heterozygosity ranged from 0.47 to 0.85, diverging numerically from the expected values for some loci, mainly for DIK4513. The average Ho was 0.73, slightly below expected (0.77). Such differences are further discussed in light of the HWE tests. Polymorphic information content (PIC) ranged from 0.57 (BM1237) to 0.88 (DIK5183). Combined with the very small non-exclusion probabilities (Table 1), these results indicate the suitability of this set of markers for studying genetic diversity in the Guzerá breed.

### 3.2. Global Hardy–Weinberg Equilibrium Tests

Considering the meta-population, significant (*p* < 0.05) results were obtained from the HWE test for each locus (Supplementary Table S2). Heterozygote deficiency was observed in nine loci (BM7169, DIK5382, DIK4383, NLBCMK13, DIK4593, DIK5307, DIK5183, DIK4513, and DIK2279). Among the possible causes are non-random mating, such as inbreeding or unidirectional gene flow caused by migration, mainly through artificial insemination (AI); or the linkage disequilibrium between the microsatellite and a locus under selection [42,43].

### 3.3. F-Statistics and Genetic Distances

Table 2 presents an average of 21 loci F-statistics [32] for both approaches, with the subpopulations considered according to farm or lineage. Fit values suggest a low fixation index, revealing a large amount of genetic diversity in the Guzerá herds sampled. The F_st_ and F_is_ values obtained in this study were lower than those reported previously in the pedigree data analysis of Guzerá animals registered in the 1994–1998 period: F_st_ = 1.75 and F_is_ = 1.37 [9]. These differences could be attributed to differences in methodology between the studies. However, despite the F_is_ and F_st_ values found in this study being below 0.05, it must be highlighted that there are situations in which F_st_ estimates lower than 0.05 do not necessarily imply negligible genetic differentiation [31]. These values support the hypothesis that these subpopulations have been going through a process of genetic differentiation in the approaches used. One hypothesis to explain the differences in the F_st_ would be that, before AI, most farmers tended to use their own or few lineages preferentially, which resulted in lower genetic diversity. This process was reverted by the introduction of AI as well as by the availability of sires of accurate genetic merit, from other herds or lineages, since the release of the first sire summary with the consequent higher gene flow (migration) between herds [7,9].

**Figure 1:**
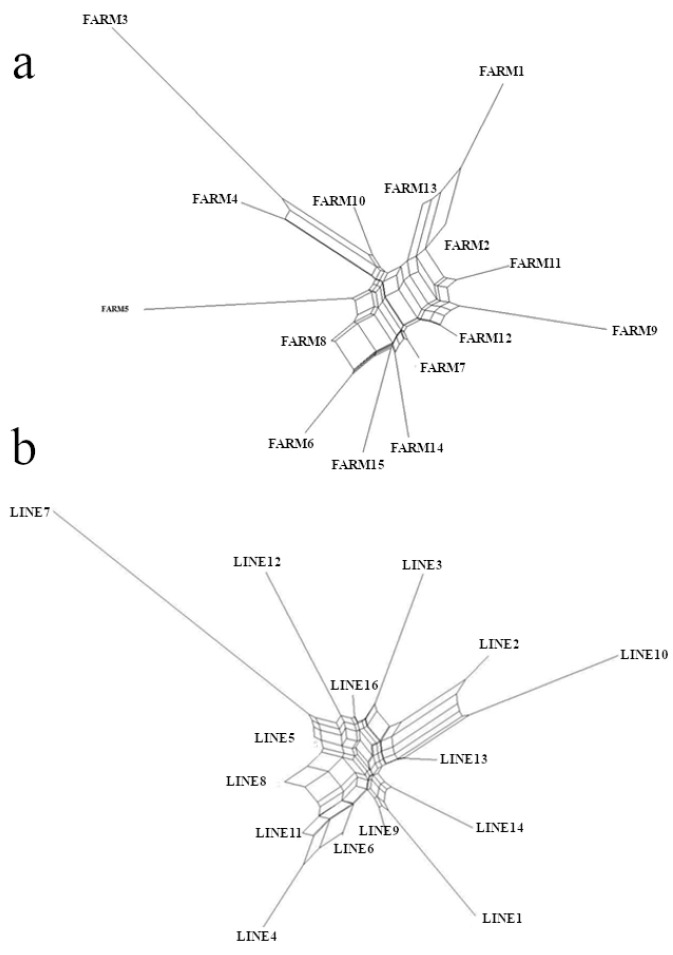
Dendrogram summarizing genetic relationships among Guzerá farms (**a**) and selection lines (**b**), using lineages F_st_ (F distances) generated by Poptree2 for the Guzerá meta-population in Minas Gerais state, Brazil.

The approach considering lineages as subpopulations showed the impact of the distinct lineages in the population structure. The F_is_ was slightly lower (−0.007) and the F_st_ was slightly higher (+0.007) than those for farms. The F_st_ for the lineage approach was 0.034, compared to 0.027 for subpopulations grouped according to farm (Table 2). Table 3 presents the F_is_ values for each farm subpopulation and pairwise F_st_, as well as the genetic distances between farms and lineages. These values are displayed graphically in Figure 1.

Estimated values for F_is_ using the farm approach ranged from −0.068 to 0.077. This indicates that genetic variability within subpopulations varied among farms, and these differences allow the reconstruction of historical aspects of the Guzerá breed in Brazil. Some farms developed their own lineages and kept them closed over time, avoiding the use of animals from other lineages in their breeding. FARM6, for example, developed LINE7. It is the only farm selecting exclusively for milk production and has been completely closed since the beginning of the 20th century. Nonetheless, LINE7, being one of the oldest, contributed to all the dual-purpose farms and even to some of the beef farms. Out of the 15 lineages, 5 were selected only for beef traits, each one coming from a specific farm: FARM1 (LINE4), FARM2 (LINE6), FARM3 (LINE10), FARM4 (LINE1), and FARM5 (LINE12).

Farms selecting for dual-purpose were originally beef farms or newly formed. For example, FARM11 (LINE9) was also closed for many years and, only from the end of the 20th century, started to use bulls of different lineages. On the contrary, FARM7 and FARM15 were recently formed by animals of different lineages. In the dual-purpose selection group, the correspondence between farms and lineages was far less strict. These historical aspects affected both the genetic diversity, F_is_, F_st_, and genetic distances.

For example, some farms (FARM6, FARM10, and FARM14) showed lower levels of heterozygosis, probably resulting from the intensive or exclusive use of their own sires or a small number of sires from few origins. At the same time, other farms (FARM1, FARM7, FARM11, and FARM15) showed high heterozygosis, attributable to the broad use of planned mating and/or semen from animals of different origins or lineages.

Estimated values for F_is_ using the lineage approach ranged from −0.109 to 0.061. This indicates that genetic diversity also varied among lineages. Some lineages were developed as part of the selection strategy of farms. In those cases, higher F_is_ were observed for both the farms and the lineages developed within them. For example, some lineages (LINE3, LINE6, and LINE7) showed higher F_is_ and lower heterozygosity, probably resulting again from the intensive or exclusive use of their own sires or a small number of sires from few origins.

Other lineages (LINE8, LINE9, and LINE14) showed high heterozygosity. LINE8 originated from LINE7, hence the high genetic similarity between them, and it has also been composed of other lineages resulting in its high heterozygosity. LINE14 came from FARM15, which contains a herd recently formed from a large genetic basis, hence its high degree of heterozygosity.

The pairwise F_st_ estimates are shown in Table 3. Pairwise F_st_ ranged from 0.0029 (FARM15 × FARM1) to 0.048 (FARM6 × FARM3) using the farm approach. They ranged more widely, from 0.007 (LINE8 × LINE11) to 0.087 (LINE7 × LINE10), using the lineage approach. In general, higher average F_st_ values were estimated for beef lineages. This means that less gene flow has occurred (pairwise F_st_), both in the meta-population and in the whole (average F_st_). This contrasts with the higher gene flow among dual-purpose lineages expressed by the lower average F_st_ estimates. One exception can be observed regarding LINE6 (FARM2), which has the lowest pairwise F_st_ average among the beef lineages (0.018 in the lineage and 0.015 in the farm approaches). Guzerá beef cattle are more extensively bred, generally using little artificial insemination and thus less exchange of genetic material between herds or lineages. However, considering the results of this study, beef herds kept a larger amount of the genetic diversity in Guzerá breeds. 

In general, the lower average F_st_ estimates for the dual-purpose selection lines reveal high genetic variability. This is probably a result of the wide genetic basis for these subpopulations. One clear exception was observed among the dual-purpose selection lines: the average F_st_ estimated for LINE7 was 0.052, the highest value among all the subpopulations considering dairy, dual-purpose, and beef selection lines. This subpopulation was closed for many generations, and this F_st_ value reinforces the available information on the breeding policy for this lineage [44]. It should be emphasized that this lineage was selected from one of the first Guzerá animals introduced into Brazil in the early 20th century. Fixation index values were less variable among dual-purpose herds, which indicates that gene flow was greater among farms than among lineages. Thus, some fraction of these herds is most likely kept closed.

**Figure 2:**
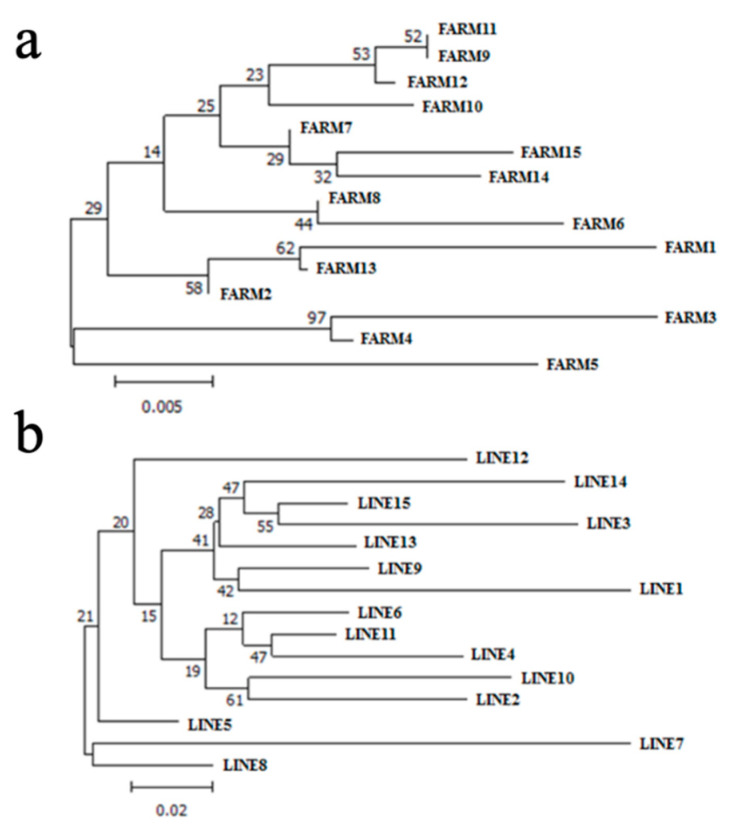
Dendrogram summarizing genetic relationships among Guzerá farms (**a**) and lineages (**b**), using Nei’s standard genetic distances generated by Poptree2 for the Guzerá meta-population in Minas Gerais state, Brazil.

Nei’s standard genetic distances ranged from 0.05 (FARM8 ×FARM6, or FARM11 × FARM7) to 0.031 (FARM9 × FARM3, or FARM6 × FARM3) for the farm grouping approach (Figure 2a). In the lineage approach (Figure 2b), these distances ranged from 0.05 (LINE8 × LINE13) to 0.53 (LINE7 × LINE10). On average, LINE7 was placed on a separate branch of the dendrogram (Figure 2b). It is the most genetically distant subpopulation, despite having contributed to all dual-purpose subpopulations. It was especially distant from LINE4 and LINE10, both of which were selected for beef purposes. The values obtained for the genetic distances were generally in agreement with the F-statistics results, validating the conclusions about population structure and gene flow observed among dual-purpose herds (Figure 2).

In the dendrogram, LINE4, LINE6, and LINE11 were clustered to the same branch. Indeed, LINE11 was derived mainly from LINE4, LINE8, and LINE6 (Figure 1b). In addition, LINE4 and LINE6 were derived from geographically close farms that exchange some genetic material. The same has occurred with LINE9, LINE1, and LINE14. LINE9 contributed to LINE1, LINE14, LINE12, and LINE5. In addition, LINE12 also contributed to LINE5 and they both received contributions from LINE7. LINE3 introduced genetic material from LINE15. LINE10 and LINE2, which are geographically close, exchanged genetic material more frequently. Therefore, the dendrogram shown in Figure 2 reflects the historical register of the Guzerá lineage formation.

Table 4 shows the results of some studies that evaluated the genetic diversity of bovine breeds. These studies were selected based on two criteria: publication after 2000 and sample size greater than 100. There is an interesting relationship between the sample size and the number of microsatellite markers used in the present study. Despite this comparison, it is important to highlight the differences in experimental design, analysis, and result presentation/interpretation. Consequently, the comparison among the results must be interpreted carefully. The 744 animal sample size is larger than the samples of all studies except one [45]. However, the number of microsatellite markers used in that study was smaller than the number used in the present study. The F_st_ values obtained using the farm (0.025) and lineage (0.034) subpopulations were lower than almost all the results shown in Table 4. Comparing heterozygosity, the values obtained for the Guzerá breed (Ho = 0.73 and He = 0.77) in the present study were generally greater than those obtained for all breeds used in the studies shown in Table 4. These results suggest that the Guzerá breed has a greater genetic diversity, a lower degree of population subdivision, and a lower inbreeding level than the breeds analyzed in these other studies. Interestingly, the results obtained in the current study are similar to those obtained using microsatellites and single nucleotide polymorphisms in other Brazilian breeds [15,22]. 

### 3.4. Quantifying Contributions to Diversity

The contribution of each subpopulation to the overall genetic diversity is presented in Table 5. Some lineages contributed more to the increase of overall diversity because of their internal diversity. This group includes LINE15, LINE11, LINE6, and LINE8. On the other hand, some lineages had very low internal diversity, decreasing the overall genetic diversity. LINE7 is an example of a subpopulation with very low internal diversity, presenting the largest loss of genetic diversity (Loss-gain ratio: 0.1056). This herd was established using a small number of animals and, since then, has remained closed. Additionally, it is the only herd that did not have genetic flow from other farms and/or subpopulations. The mean distance between subpopulations is another feature contributing to the overall genetic diversity. Thus, LINE7 and LINE10 could be contributing to the increase in the mean distance between subpopulations, since they are the most closed lineages.

### 3.5. Numbers of Clusters and Lineages

**Figure 3:**
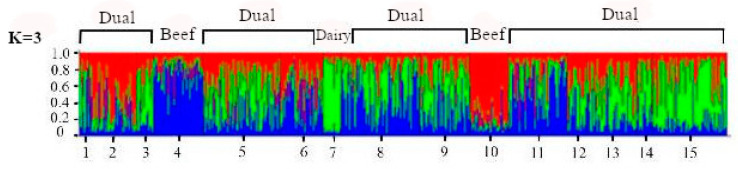
Histogram showing the population structure for the 15 lineages of the Guzerá cattle meta-population in Minas Gerais state, Brazil, generated using the model-based STRUCTURE software. Each animal is represented by a single vertical line divided into K colors, where K is the number of clusters assumed and the colors show the estimated individual proportions of cluster membership. K = 3: cluster 1—red (beef cattle); cluster 2—dark blue (beef cattle); and cluster 3—green (dual-purpose).

An interesting result was obtained with respect to meta-population structure (Figure 3). If lineages were used as subpopulations, K = 3 was the most probable one, separating the beef (mainly LINE4 and LINE10) from the dual-purpose (mainly LINE3, LINE5, LINE8, LINE9, LINE13, LINE14, and LINE15) and dairy (LINE7) lineages. LINE7 is the only exclusively dairy lineage and it was used to generate all the dual-purpose cattle. The dual-purpose lineages, shown in Figure 3, have contributions from the green component, which corresponds to the dairy lineages, reinforcing the historical contribution of LINE7 to the formation of the dual-purpose. Beef cattle were separated into two subpopulations: one, consisting of lineages from closed herds with little gene flow; the other, consisting of lineages from less closed herds that strongly contributed to the dual-purpose herds (LINE4 and LINE10, respectively). The inclusion of LINE8 and LINE11 in one of the dual-purpose cattle clusters was also interesting. LINE11 was sequentially derived from LINE8, which was derived from LINE7. LINE11 was among the dual-purpose cattle for a period after which selection pressure was imposed for milk trait improvement but only for beef purposes, or better, for improving the developmental and weight trait performance. LINE11, LINE5, and LINE14 were all grouped in both beef and dual-purpose clusters, signaling that beef traits are also very important for meeting their selection objectives. Moreover, some dual-purpose lineages showed a strong contribution to the dairy lineage (LINE7) genetic diversity (LINE3, LINE13, LINE14, and LINE15). Therefore, indicating that these lineages can be used as genetic reservoirs for dairy genetic improvement purposes.

## 4. Conclusions

Despite the historical events that the Guzerá breed went through in the last decades, such as a strong founder effect and several population bottlenecks, the breed nevertheless preserved a good amount of genetic diversity. In the cluster analysis, it was possible to recover both the origin of the lineages and the selection purposes. Reasonably, in the best model (K = 3), dual-purpose selection lines clustered with the beef selection lines from which they originated. Also, gene flow between the different lineages was detected in the clusters formed. Modeling the genetic structure of the Guzerá meta-population helped to understand the impact of planned crosses on the genetic diversity and the distribution of the genetic diversity among lineages selected for different purposes (beef, dairy, and dual-purpose). The separation of animals for a new selection purpose, if not implying a stronger selection pressure, decreases the risk of loss of diversity. Taken together, these results suggest that, despite the population structure produced by selection strategies based originally on relatively closed farms/lineages, the amount of genetic diversity conserved in the Guzerá breed is high. In particular, beef farms/lineages emerge as a reservoir for both genetic selection and conservation programs.

## Figures and Tables

**Table 1:** Combined analysis of 21 microsatellite loci of the Guzerá cattle at Minas Gerais, Brazil.

Number of individuals:	723
Number of loci:	21
Mean number of alleles per locus:	13.67
Mean proportion of individuals typed:	0.9187
Mean expected heterozygosity:	0.7746
Mean polymorphic information content (PIC):	0.7473
Combined non-exclusion probability (first parent):	1.17 × 10^−5^
Combined non-exclusion probability (second parent):	5.64 × 10^−9^
Combined non-exclusion probability (parent pair):	8.41 × 10^−15^
Combined non-exclusion probability (identity):	2.16 × 10^−24^
Combined non-exclusion probability (sib identity):	1.63 × 10^−9^

**Table 2:** F-statistics (F_is_, F_st_, F_it_) means, standard errors (SE), and 95% confidence intervals obtained for the Guzerá dairy metapopulation, with the subpopulations defined as farm sampled (on the left) or as the lineage (on the right).

		F-Statistics					
		Farms as Subpopulations			Lineages as Subpopulations		
	Limits	F_is_	F_st_	F_it_	F_is_	F_st_	F_it_
Value (SE)	-	0.029 (0.022)	0.027 (0.002)	0.055 (0.021)	0.022 (0.022)	0.034 (0.002)	0.055 (0.021)
95% confidence interval	Min:	−0.007	0.023	0.021	−0.014	0.029	0.021
	Max:	0.076	0.031	0.100	0.070	0.038	0.100

SE: Standard error. Min: minimum and Max: Maximum.

**Table 3:** Lineage F_is_, farm F_is_, pair-wise lineages F_st_ (above diagonal), and pair-wise farms F_st_ (below diagonal). Average values of F_st_ and D’s for each farm are presented below the matrix (italic), while average values of F_st_ and D’s for each lineage (LINE) are presented above the matrix.

	Lineage	LINE1	LINE2	LINE3	LINE4	LINE5	LINE6	LINE7	LINE8	LINE9	LINE10	LINE11	LINE12	LINE13	LINE14	LINE15	
FARMS																	
FARM1		0.018 0.003	0.019	0.031	0.017	0.010	0.013	0.058	0.010	0.008	0.031	0.010	0.035	0.013	0.024	0.008	LlNE1
FARM2		0.013	0.008 0.033	0.019	0.036	0.020	0.017	0.058	0.024	0.022	0.027	0.025	0.024	0.019	0.022	0.019	LINE2
FARM3		0.040	0.025	0.061 0.020	0.035	0.011	0.019	0.060	0.014	0.018	0.039	0.020	0.030	0.017	0.028	0.009	LINE3
FARM4		0.032	0.015	0.025	0.003 0.022	0.024	0.014	0.066	0.018	0.022	0.039	0.009	0.032	0.030	0.023	0.027	LINE4
FARM5		0.032	0.016	0.042	0.023	0.027 0.031	0.013	0.025	0.013	0.013	0.027	0.012	0.013	0.014	0.011	0.013	LINE5
FARM6		0.036	0.022	0.048	0.027	0.023	0.037 0.048	0.052	0.011	0.012	0.025	0.009	0.020	0.013	0.018	0.014	LINE6
FARM7		0.025	0.013	0.031	0.017	0.014	0.013	0.038 0.006	0.030	0.041	0.087	0.044	0.062	0.047	0.063	0.029	LINE7
FARM8		0.025	0.013	0.028	0.019	0.014	0.009	0.011	−0.030 0.033	0.014	0.034	0.007	0.016	0.018	0.013	0.014	LINE8
FARM9		0.017	0.011	0.031	0.017	0.035	0.016	0.008	0.010	−0.019 0.018	0.040	0.011	0.021	0.012	0.013	0.009	LINE9
FARM10		0.029	0.014	0.030	0.017	0.019	0.021	0.008	0.014	0.012	0.020 0.046	0.034	0.042	0.029	0.036	0.034	LINE10
FARM11		0.017	0.009	0.037	0.016	0.029	0.019	0.008	0.013	0.016	0.013	0.028 −0.068	0.019	0.019	0.014	0.016	LINE11
FARM12		0.024	0.014	0.040	0.020	0.023	0.014	0.008	0.011	0.009	0.011	0.009	0.031 0.024	0.020	0.039	0.014	LINE12
FARM13		0.010	0.006	0.024	0.016	0.010	0.012	0.011	0.009	0.005	0.013	0.006	0.009	0.032 0.017	0.016	0.011	LINE13
FARM14		0.003	0.014	0.039	0.018	0.026	0.014	0.006	0.009	0.023	0.014	0.018	0.015	0.008	−0.109 0.077	0.011	LINE14
FARM15		0.0029	0.018	0.043	0.024	0.035	0.016	0.010	0.013	0.021	0.017	0.020	0.015	0.009	0.017	0.005 −0.043	LINE15
		FARM1	FARM2	FARM3	FARM4	FARM5	FARM6	FARM7	FARM8	FARM9	FARM10	FARM11	FARM12	FARM13	FARM14	FARM15	
Average Fst		0.021	0.025	0.025	0.028	0.016	0.018	0.052	0.017	0.018	0.037	0.018	0.028	0.020	0.024	0.016	Lineage
		0.024	0.015	0.035	0.020	0.024	0.021	0.013	0.014	0.017	0.017	0.017	0.016	0.011	0.016	0.021	Farm
Average D’s		0.20	0.19	0.21	0.20	0.12	0.13	0.33	0.14	0.14	0.25	0.14	0.22	0.14	0.17	0.13	Lineage
		0.171	0.108	0.241	0.151	0.190	0.144	0.103	0.108	0.171	0.118	0.126	0.110	0.112	0.134	0.142	Farm

**Table 4:** Results of the evaluation of genetic diversity in other breeds.

Breed	Number of Individuals	Number of Markers	Mean Expected Heterozygosity (He)	Mean Observed Heterozygosity (Ho)	F-Statistics	Reference
Ethiopian Cattle	351	30	0.726	0.674	F_st_ = 0.013	[16]
Creole Cattle	857	19	0.738	0.718	F_is_ = 0.028	[45]
Korean Cattle Chinese Cattle Japanese Black Cattle European Holstein	200	13	0.728 0.744 0.471 0.693	0.721 0.745 0.515 0.753	F_st_ = 0.109	[46]
Sahiwal Hariana Deoni	136	20	0.61 0.66 0.70	0.42 0.53 0.59	F_st_ = 0.067	[47]
Japanese Black Cattle	252	20	0.618	0.623	F_st_ = 0.151	[48]
Jersey	223	12	0.643	0.6355	F_st_ = 0.013 ^†^ F_st_ = 0.035 ^††^	[49]
Burlina Cattle	279	24	0.69	0.63	F_st_ = 0.036	[50]
Pirenaica Betizu Terreña Monchina	302	11	0.688 0.715 0.747 0.762	0.682 0.651 0.737 0.757	F_st_ = 0.041	[51]
Caracu; Criolo Lageano; Curraleiro; Mocho Nacional and Pantaneiro; Holstein and Jersey	623	22	0.793	0.695	F_st_ = 0.061	[15]
Nellore; Gyr and Guzerá	292	22	0.748	0. 645	F_st_ = 0.040	[15]
Vietnamese indigenous cattle	410	27	0.760	0.680	F_st_ = 0.04	[52]

^†^ Between parishes; ^††^ Between Farms.

**Table 5:** Genetic diversity (GD), within-subpopulation contribution to GD (Internal_Diversity), between-subpopulations contribution to GD (Mean_Distance), and total contribution to GD (Loss/Gain) after hypothetically removing each subpopulation.

Lineage	GD	Internal Diversity	Mean Distance	Loss/Gain
LINE1	0.7749	0.0046	−0.0358	−0.0312
LINE2	0.7731	−0.1360	−0.1267	−0.2627
LINE3	0.7750	0.0484	−0.0671	−0.0186
LINE4	0.7759	0.2753	−0.1740	0.1013
LINE5	0.7764	−0.0883	0.2467	0.1585
LINE6	0.7745	−0.2157	0.1370	−0.0787
LINE7	0.7759	**0.5530** *	**−0.4474** *	0.1056
LINE8	0.7751	−0.1929	0.1903	−0.0026
LINE9	0.7747	−0.1712	0.1201	−0.0511
LINE10	0.7739	0.2400	−0.3954	−0.1554
LINE11	0.7746	−0.2397	0.1690	−0.0707
LINE12	0.7746	0.0260	−0.0925	−0.0665
LINE13	0.7759	0.0362	0.0609	0.0971
LINE14	0.7759	0.1356	−0.0359	0.0997
LINE15	0.7739	−0.3457	0.1868	−0.1589

* The higher values found for Internal Diversity and Mean distance are indicated in bold.

## Supplementary Materials

The following are available online at https://www.mdpi.com/article/10.3390/ani11041125/s1, Table S1: Sample distribution according to selection purpose and farms; Table S2: Microsatellite loci description.
