# Supplementary material for: Genetic Diversity and Population Genetic Structure of a Guzerá (Bos indicus) Meta-Population
Source: Animals (Basel). 2021 Apr 14;11(4):1125. doi: 10.3390/ani11041125 (PMC8071051; doi:10.3390/ani11041125)
Supplement: Supplementary file 1 [file animals-11-01125-s001.pdf]

Table S1. Sample distribution according to selection purpose and farms.

Selection Purpose	Farms	N
Beef	1 to 5	223
Dairy	6	54
Dual	7 to 15	446
**Total**	-	723

Table S2. Microsatellite *loci* description.

Locus	BTA	Chromosomal position	k	N	HO	HE	PIC	NE-1P	NE-2P	Score test (p-value)	X^2 test (p-value)	F(Null)
ILSTS093	6	0.00	15	662	0.83	0.82	0.80	0.51	0.34	0.9880	0.1037	-0,02
MNB-208	6	60.21	10	719	0.80	0.78	0.75	0.60	0.42	0.9962	0.6442	-0,01
BM1237	10	24.70	15	681	0.57	0.59	0.57	0.78	0.60	0.4720	0.4998	0,02
BMS2614	10	109.39	15	731	0.75	0.77	0.74	0.60	0.42	0.2509	0.0000**	0,01
BM7169	11	50.31	19	653	0.75	0.77	0.73	0.62	0.44	**0.0172***	**0.0268***	0,01
RM150	11	70.14	9	724	0.79	0.80	0.77	0.56	0.38	0.8609	0.1472	0,01
BMS2252	12	14.36	13	619	0.85	0.81	0.79	0.54	0.37	0.9764	0.0421*	-0,03
JAB8	15	31.21	11	658	0.78	0.77	0.75	0.60	0.42	0.8967	0.6467	-0,01
DIK5382	15	101.07	13	728	0.75	0.83	0.81	0.51	0.34	0.0000**	0.0000**	0,05
DIK4383	17	78.07	8	713	0.72	0.79	0.76	0.59	0.41	0.0000**	0.0000**	0,04
NRDIKM004	20	0.63	10	671	0.72	0.73	0.69	0.67	0.50	0.8997	0.7186	0,01
NLBCMK13	20	30.11	27	734	0.75	0.81	0.79	0.53	0.36	0.0000**	0.0000**	0,04
DIK4593	21	1.04	9	564	0.60	0.67	0.64	0.72	0.54	0.0080*	0.0012**	0,05
MNB-88	21	45.19	11	705	0.76	0.79	0.75	0.59	0.42	0.0025**	0.1297	0,02
MNS-20	22	21.92	9	661	0.82	0.83	0.81	0.51	0.34	0.6554	0.2867	0,00
DIK5307	22	88.10	14	730	0.70	0.73	0.69	0.67	0.49	0.0000**	0.0000**	0,01
DIK5183	25	3.58	25	617	0.82	0.89	0.88	0.36	0.22	0.0000*	**0.0052***	0,04
DIK4513	26	23.57	18	678	0.47	0.79	0.76	0.58	0.40	0.0000*	0.0000**	0,26
DIK2279	26	61.89	11	715	0.67	0.78	0.75	0.60	0.42	0.0000*	0.0000**	0,08
DIK5300	28	14.75	15	662	0.81	0.79	0.77	0.56	0.39	0.5847	0.0001**	-0,01
DIK1143	28	61.65	10	728	0.71	0.73	0.70	0.67	0.48	0.9748	0.1141	0,01
Average			**13.7**	**684**	**0.73**	**0.77**	**0.75**	**0.59**	**0.41**			0,03

Note: BTA – Bos taurus chromosome to which each marker maps; K – number of alleles per loci; N – number of individuals test per loci; HO – observed heterozygosity; HE – expected heterozygosity; PIC – polymorphic information content; NE-1P – non-exclusion probability considering one possible parent; NE-2P – non-exclusion probability considering two possible parents. HWE test was tested using the Score test (Rousset 2008) and exact P-value of this test was established accordingly (Guo & Thompson 1992).
